# Mesenchymal stem cells and cancer therapy: insights into targeting the tumour vasculature

**DOI:** 10.1186/s12935-021-01836-9

**Published:** 2021-03-08

**Authors:** Surendar Aravindhan, Sura Salman Ejam, Methaq Hadi Lafta, Alexander Markov, Alexei Valerievich Yumashev, Majid Ahmadi

**Affiliations:** 1 Department of Pharmacology, Saveetha dental College and hospital, Saveetha institute of medical and technical sciences, Chennai, India; 2 Department of Pathology, College of Medicine, University of Babylon, Babylon, Iraq; 3 Iraqi Ministry of Education, Baghdad, Iraq; 4 Tyumen State Medical University, Tyumen, Russian Federation; 5 Department of Prosthetic Dentistry, Sechenov First Moscow State Medical University Moscow, Moscow, Russian Federation; 6 Stem Cell Research Center, Tabriz University of Medical Sciences, Tabriz, Iran

**Keywords:** Mesenchymal stem cells, Tumor microenvironment, Anti-angiogenesis, Tumor therapy

## Abstract

A crosstalk established between tumor microenvironment and tumor cells leads to contribution or inhibition of tumor progression. Mesenchymal stem cells (MSCs) are critical cells that fundamentally participate in modulation of the tumor microenvironment, and have been reported to be able to regulate and determine the final destination of tumor cell. Conflicting functions have been attributed to the activity of MSCs in the tumor microenvironment; they can confer a tumorigenic or anti-tumor potential to the tumor cells. Nonetheless, MSCs have been associated with a potential to modulate the tumor microenvironment in favouring the suppression of cancer cells, and promising results have been reported from the preclinical as well as clinical studies. Among the favourable behaviours of MSCs, are releasing mediators (like exosomes) and their natural migrative potential to tumor sites, allowing efficient drug delivering and, thereby, efficient targeting of migrating tumor cells. Additionally, angiogenesis of tumor tissue has been characterized as a key feature of tumors for growth and metastasis. Upon introduction of first anti-angiogenic therapy by a monoclonal antibody, attentions have been drawn toward manipulation of angiogenesis as an attractive strategy for cancer therapy. After that, a wide effort has been put on improving the approaches for cancer therapy through interfering with tumor angiogenesis. In this article, we attempted to have an overview on recent findings with respect to promising potential of MSCs in cancer therapy and had emphasis on the implementing MSCs to improve them against the suppression of angiogenesis in tumor tissue, hence, impeding the tumor progression.

## Introduction

Given the importance of cancer, research on timely diagnosis and effective treatment in cancer field is on the fast track [1, 2]. Old different cancer therapies, such as radiotherapy and chemotherapy are both harmful to normal cell and many other side effects [3, 4]. These limitations have led to the spread of studies on efficient therapeutic strategies specifically targeting malignancies [5, 6]. One type of stem cells for therapeutic approaches of cancer is mesenchymal stem cells (MSCs). Numerous studies have previously demonstrated the potential of MSC therapy for various diseases [7]. MSCs have different advantages like simplicity of expression and differentiate into various cell types, which have caused widespread using MSCs for therapeutic applications to treat cancer and other disease. Some MSCs can be extracted from different types of tissues such as brain, kidney, and heart. In addition, these stem cells have the ability of development into mesenchymal lineages and self-renewal. MSC therapy is a sub-type of cell therapy and regenerative medicine. Other stem cells such as induced pluripotent stem cells (iPS) and pluripotent embryonic stem cells (ES) have some disadvantages, including teratoma formation and ethical concerns. Therefore, considering that MSCs do not have this limitations, they are frequently used for cancer therapy [8]. One of the important reason for tumor cells proliferation is tumor angiogenesis [9]. Therefore, given that MSCs can inhibit expression of anti-angiogenesis factors, attentions for utilization of targeted MSCs therapy of cancers by inhibition of angiogenesis have been raised [10].

This review paper endeavoured to go through the recent findings with regard to promising potential of MSCs in cancer therapy and have emphasis on the implementing MSCs to improve them against the suppression of angiogenesis in tumor tissue.

## Biology of MSC and its implication in tumors

### Origin and characteristics of MSCs

In addition to bone marrow, MSCs have been extracted from multiple tissues, such as adipose tissues, skeletal muscle, dental pulp, placenta, synovial membranes and umbilical cord [11]. Some important advantages of MSCs are easily accessible, self-renewable, culturally expandable in vitro, and being multipotent [12]. Some other biological characteristics, including immune regulatory, potential of differentiation, and secretion of trophic factors that help tissue remodelling have caused MSCs suitable for cancer therapy [13, 14]. MSCs operate immunomodulatory functions by different ways. They express low levels of costimulatory CD80, CD86, CD40 proteins and major histocompatibility complex (MHC) class I and no MHC class II molecules. MSCs have a role in upregulating the secretion of IL-4 and IL-10 (immune suppression cytokines). MSCs secrete different factors in site of injury that have ability in regenerative processes by inducing angiogenesis, modulating immune system, and protecting cells from apoptotic cell death [15].

Mobilization from the bone marrow and other organs is the very necessary role for MSC homing to tumors. Endogenous MSCs have been demonstrated to mobilize from the bone marrow and other tissues to the peripheral tissues through various injury conditions, such as normoxia, inflammatory conditions, and hypoxia [16]. It has been revealed that a normal role of MSC is the potential to migrate and repair wounded tissue. This factor of wound healing originates with migration toward inflammatory signals caused by the injured tissue [17]. Migration is implemented through a wide range of mediators secreted by MSCs [18]. These researches conferred a validation for the development of therapeutic approaches that have role in the tumoritropic characteristics of MSCs by engineering them as delivery vehicles of antitumor compounds.

Tumor microenvironment influences on the plasticity of MSC phenotype and responses through diverse stimuli, leading to polarization of MSCs and obtaining specific characteristics [19]. Regarding the inflammatory settings, MSCs are phenotypically are categorized into pro-inflammatory MSC-1 cells (developed in response to priming by toll-like receptor (TLR) 4] and anti-inflammatory MSC-2 cells (developed in response to priming by TLR3) [20]. Priming with TLR4 stimulates the development of pro-inflammatory MSC-1 phenotype that is distinguished by higher production of inflammatory cytokines like interleukin (IL)-6 and IL-8 [21]. The pro-inflammatory MSC-1 phenotype was shown to be incapable of suppressing the expansion and proliferation of other cells [22]. The plasticity of MSCs influence on the released mediators by these cells and thereby their function.

### MSCs and tumor microenvironment

**Fig. 1:**
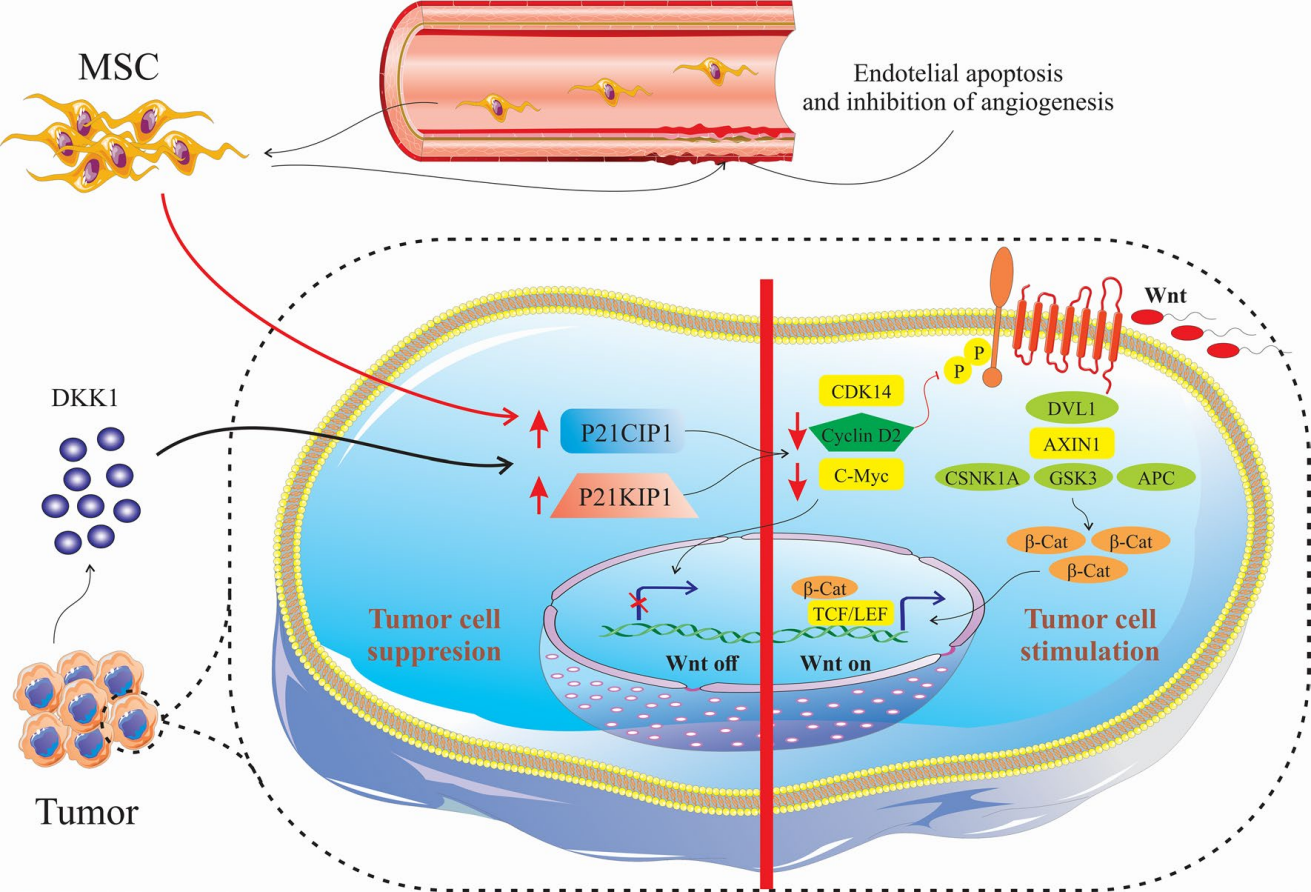
Naïve MSCs can inhibit Wnt signalling pathways through Dickkopf-related protein 1 (DKK1) released by tumor cells and subsequently downregulated c-Myc and Cyclin D2 and upregulated expression of P21CIP1 and P27KIP1, leading to tumor cells suppression. Naïve MSCs can cause apoptosis vascular endothelial cells by inhibiting angiogenesis

**Fig. 2:**
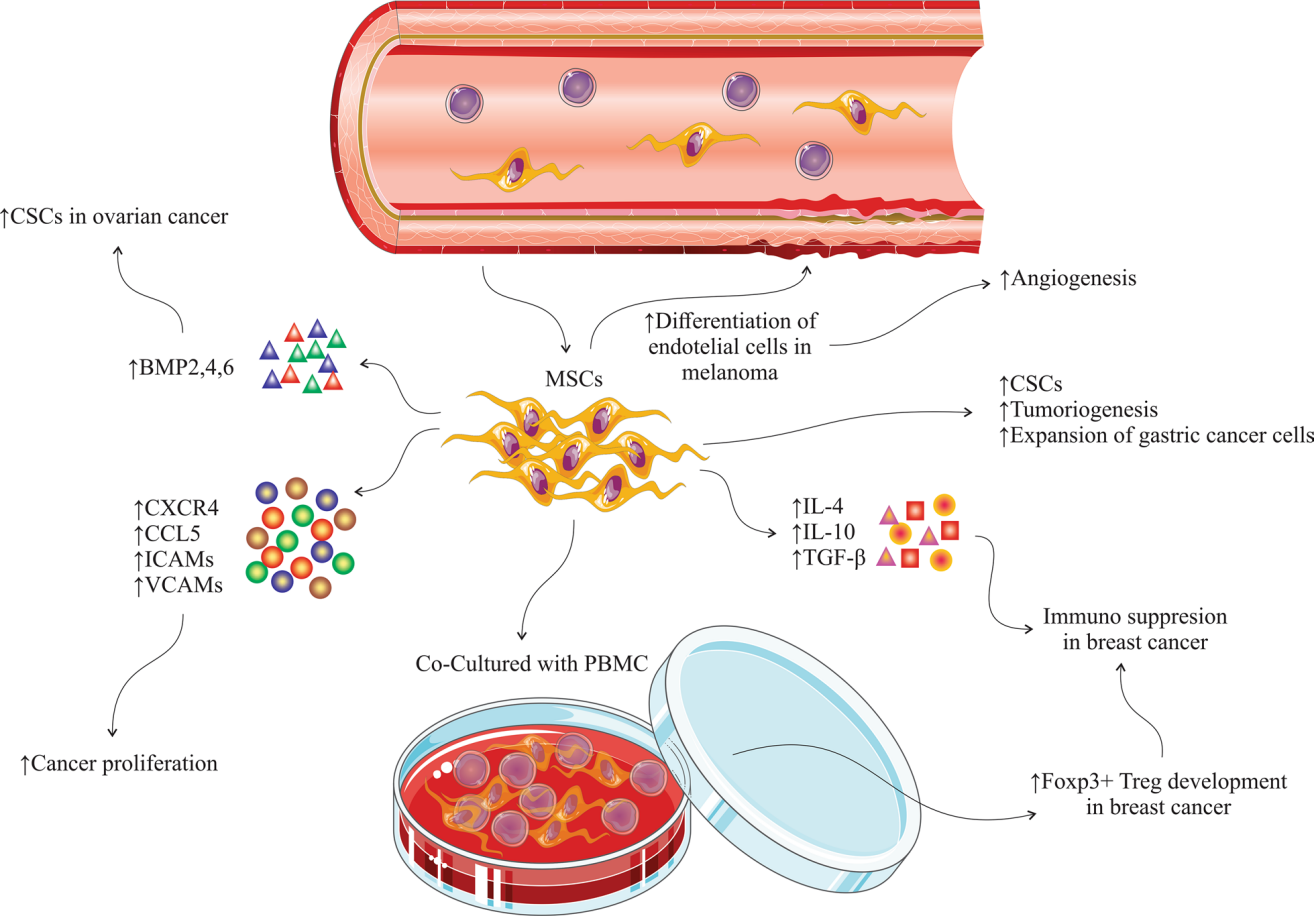
MSCs have some adverse effect on tumor cells, such as differentiation of vascular endothelial cells in melanoma, enhancing the expansion of gastric cancer cell lines, inducing of cancer stem cells (CSCs) that are involved in metastasis, tumorigenesis, and recurrence of tumors. When co-cultured with peripheral blood mononuclear cells (PBMC), MSCs from breast cancer promote the development of CD4^+^CD25^high^FOXP3^+^ regulatory T cells. MSCs derived from breast cancer tissues contain high levels of immunosuppressive mediators, such as IL-4, TGFβ and IL-10. Upregulation of bone morphogenetic proteins (BMPs), including BMP2, BMP4 and BMP6 in ovarian cancer derived MSCs can promote the development of CSCs

Numerous mediators have been identified that play critical roles in the cross-talk between MSCs, tumor microenvironment, and tumor cells (Table 1). By inducing various signalling pathways, MSCs possess different functions on the cells in tumor microenvironment. Naïve MSCs can inhibit Wnt signalling pathways through modulating the Dickkopf-related protein 1 (DKK1) released by tumor cells, and subsequently downregulating c-Myc and Cyclin D2 and upregulated expression of P21CIP1 and P27KIP1, leading to tumor cells suppression [23–25]. Naïve MSCs can cause apoptosis of the vascular endothelial cells by inhibiting angiogenesis [26] (Fig. 1). MSCs have some adverse effect on tumor cells, such as differentiation of vascular endothelial cells in melanoma [27], enhancing the expansion of gastric cancer cell lines [28], inducing cancer stem cells (CSCs) that has been associated with increased metastasis, tumorigenesis, and recurrence of tumors [29]. Additionally, MSCs release chemokines, such as CXCR4 [30], CCL5, intracellular adhesion molecules (ICAMs) and vascular cell adhesion molecules (VCAMs) [31, 32]. MSCs isolated from mouse lymphomas (L-MSCs) release CCL2 and promote cancer cell proliferation and also recruitment of immunosuppressive cells, including CD11b+Ly6G+neutrophils, F4/80+ macrophages to lymphoid tissues [33]. Previous studies demonstrated that MSCs from breast cancer, when co-cultured with peripheral blood mononuclear cells (PBMC), resulted in development of regulatory T cells [34]. Moreover, MSCs derived from breast cancer tissues generate high amount of immunosuppressive mediators such as IL-4, transforming growth factor (TGF)-β and IL-10 [34]. Increased levels of bone morphogenetic proteins (BMPs), including BMP2, BMP4, and BMP6 in ovarian cancer derived-MSCs can promote the development of CSCs. BMP2 was detected to be involved in promoting the phospho-SMAD 1/5 protein levels in the SKOV3 cells (ovarian cancer cell line) and BMP2 inhibitor (Noggin) supressed this process. [35]. In fact, BMP2 activates phospho-SMAD signalling in ovarian cancer and is involved in triggering the epithelial-to-mesenchymal transition [36] (Fig. 2).

**Table 1:** Cytokines and mediators secreted from MSCs during cross-talk with tumor cells responsible for homing, growth, and stemness in tumor cells

Effect	Mediator type	Mediator	References
Homing of MSC in tumor	Cytokine	TNF-α	[132, 133]
		IFN-γ	[132]
		IL-1β	[132]
		IL-6	[29]
		IL-8	[134]
	Chemokine	SDF-1/CXCR4	[135, 136]
		MCP-1	[137]
		GRO-α	[134]
	Growth factor	TGF-β	[138]
		PGF	[139]
		PDGF	[132]
		HGF	[132]
MSC-mediated tumor growth (metastasis and proliferation)	Cytokine	IL-6	[38, 140]
		IL-10	[140]
		TNF-α	[140]
	Chemokine	CCL5	[31, 141]
		CXCL1	[142]
		CXCL2	[142, 143]
		CXCL3	[142]
		CXCL5	[142]
		CXCL6	[142]
		CXCL8	[116, 142]
		CCL2	[142]
		CCL8	[142]
		CCL20	[142]
	Growth factor	IGF-1	[144]
		TGFβ1	[145]
		HGF	[146]
MSC-mediated cancer cell stemness	Cytokine	IL-1α	[147]
		IL-1β	[147]
		IL-6	[147, 148]
	Chemokine	CXCL1	[147]
		CXCL8	[147]
		CXCL1	[29]
		CXCL5	[29]
		CXCL6	[29]
		CXCL7	[29]
		CCL5	[141]
	Growth factor	BMP4	[35]
		PGE2	[147]

*MSC* Mesenchymal stem cell, *TNF* tumor necrosis factor, *IFN* interferon, *IL* interleukin, *SDF* stromal cell-derived factor, *MCP* monocyte chemoattractant protein, *GRO* growth-regulated oncogene, *TGF* transforming growth factor, *PGF* placental growth factor, *PDGF* platelet-derived growth factor, *HGF* hepatocyte growth factor, *BMP4* bone morphogenetic protein, *IGF-1* insulin-like growth factor-1, *PGE2* prostaglandin E2

The communication of MSCs with tumor microenvironment might either supress or promote the tumor progression. Although most of researches aiming to exert MSCs in cancer therapy have focused on the tumor suppressor properties of MSCs, these cells may also promote tumor advancement. In vivo and in vitro experiments revealed that human MSCs were able to enhance the metastasis and growth of tumor cell in a mice model of osteosarcoma [37]. Additionally, MSCs were detected to promote the cancerous behaviour of tumor cells in ovarian cancer [38], colon cancer [39], and gastric cancer [40]. MSCs accelerate the tumor progression mainly through enhancing metastasis, contributing to epithelial–mesenchymal transition, and disturbing the immune surveillance. MSCs might show adverse effects during tumor therapy based on the number of MSCs injected, source or origin of MSC, differentiation level of MSC, and tumor type. As a consequence, limitations in MSC-based cancer therapy should be taken into account and further investigations needs to be performed to characterize the safety and efficacy of such therapeutic approach in tumor treatment.

## MSCs exosomes therapy in tumors

Exosomes are extracellular vesicles (EVs) that are generated in the endosomal compartment of eukaryotic cells [41]. Exosomes and other EVs can be detected in tissues and biological fluids, such as urine, blood, and cerebrospinal fluid. Exosomes predominantly contain microRNAs (miRs) and proteins surrounded with lipid bilayer membrane [42, 43]. Other RNA forms like nucleolar RNA, long noncoding RNA and ribosomal RNA and also fragments of DNA may be found in the exosomes [44]. Studies have demonstrated that secreted exosomes can be directed to other cells through proteins located at surface of cells such as tetraspanins [45]. MSCs produce exosomes that can regulate tumor cell angiogenesis, metastasis and proliferation by controlling a number of cellular pathways [46]. Additionally, MSC-derived exosomes can play paracrine role by transferring signalling molecules. MSC-derived exosomes have supporting or suppressing impact on the tumor development.

Prodrug suicide gene therapy by different MSCs can deliver the chemotherapeutic drug and activate the prodrug directly within the tumor to generate toxic products for tumor cells [47, 48]. Adipose tissue derived MSCs expressing thymidine kinase of Herpes simplex virus with ganciclovir as a prodrug TK HSV-MSC/ganciclovir system [49] was developed to generate MSCs that were able to act as a tumor-specific prodrug converting cellular vehicle for targeted chemotherapy. Additionally, MSCs engineered to express fused yeast cytosine deaminase:Uracil phosphoribosyl transferase (yCD::UPRT) with chemotherapeutic compound 5-fluorocytosine (5-FC) as a prodrug-yCD:UPRT-MSC/5–FC system in human colon cancer [47]. This system indicated the potential of adipose tissue derived MSCs as favourable delivery vehicles for prodrug converting gene. In another study, researchers prepared MSCs from various tissues and transduced them with the yCD:UPRT gene. yCD::UPRT-MSCs released prodrug mRNA and exosomes that were internalized into tumor cells, and then intracellular translation of mRNA to a protein that played a role in tumor cell death [50]. Paclitaxel (PTX) is an effective antitumor agents against cancer [51]. Kolimuthu et al. [52] loaded PTX in MSCs exosome mimetics and demonstrated cytotoxicity of MDA-MB-231 breast cancer cell lines when treated with PTX-MSC-Ems (Table 2). In a recent study, nano-sized vesicles secreted by MSC were used to encapsulate doxorubicin and determined for potential therapy in colon adenocarcinoma, resulting in introduction of MUC1 aptamer-decorated MSC-derived exosomes as a promising platform for cancer therapy [53]. Overall, such attempts to increase the efficacy of chemotherapy through employing the tumor targeting characteristic of MSCs could hopefully increase the chance of cancer therapy in different solid tumors.

**Table 2:** Strategies toward inhibition of angiogenesis to treat malignancies

Strategy		Mechanism	Therapeutic	References
Exosomal miRNAs		Exosomes can regulate tumor cell angiogenesis, metastasis and proliferation by controlling a number of cellular pathways	Exosomes have supporting or suppressing impact tumor development	[47, 48]
Inhibition of VEGF		Suppression of progression and growth of ECs	Bevacizumab, a VEGF neutralizing mAb Soluble receptor of VEGF, VEGF-TrapR1R2	[78] [81]
Inhibition of signal transduction by targeting receptor tyrosine kinases		Repression of receptor tyrosine kinases	(2-(3,4-Dihydroxyphenyl)-6,7-dimethylquinoxaline-HCl and (E)-3-(3,5-Diisopropyl-4-hydroxyphenyl)-2-[(3-phenyl-n-propyl) aminocarbonyl] acryl-onitrile)	[97]
MSC therapy	Regulation of pro-angiogenic and anti-angiogenic compounds		MSCs-based delivery of endostatin	[103]

*VEGF* vascular endothelial growth factor, *ECs* endothelial cell, *mAb* monoclonal antibody, *MSC* mesenchymal stem cells

The composition and biological activities as well as the therapeutic potential of MSC-derived EVs in cancer have been extensively reviewed by Xunian and Kalluri [54]. The virtual impression of MSC-derived exosomes on the tumor cells is still debating and might be tumor supporting or supressing effects, based on different issues, like the origin of the exosomes, components of the exosomes, and tumor type.

## Novel MSC therapy in other solid tumors

Graphene oxide (GO) is a versatile platform for bioimaging, tissue engineering, gene delivery, and drug delivery, that is functionalized with carboxylic acid [55]. The important feature of GO is large area and this feature has caused to use GO in wide range of therapeutics. Suryaprakash et al. [55] first loaded GO with two types of drugs (doxorubicin and mitoxantrone) and then loaded drug-GO complex on MSCs surface. The result of this study demonstrated MSC-GO is an useful mean to carry the drugs to the tumor cells and kill the cancer cells [55]. Human amniotic fluid mesenchymal stem cells (hAF-MSCs) obtained from second trimester or end of pregnancy by amniocentesis [56] have been used for some human life-threatening disease. Ghoizadeh et al. [57] showed that co-culture of hAF-MSCs with SKOV3 ovarian cancer cells can cause release of soluble factors and efficient anticancer effect. Tumor necrosis factor-related apoptosis-inducing ligand (TRAIL) increase apoptosis in cancer cells without side effect on normal cells [58, 59]. Different histone deacetylase inhibitors (HDACi) such as valproic acid (VPA), suberanilohydroxamic acid (SAHA) and panobinostat have effect on the malignant glioma [60, 61]. hAF-MSCs expressing TRAIL have therapeutic effect against different cancers [62]. Choi et al. [63] combined treatment with hAF-MSCs expressing TRAIL and panobinostat, which increased apoptosis and suppressed viability of glioma cells. Using PTX in the treatment of tumors has an important limitation, which is their sever dose-limiting toxicities. A study has shown that nano-engineered MSCs can be contributing in intra-tumoral distribution of the therapeutic agent [64]. Layek et al. [64] have demonstrated that combined nano-engineered MSCs with PTX anti-cancer drug can provide a better efficacy in tumor cells death than MSCs lacking nano-engineered procedures. All these investigations implicate on the application of a strengthened MSC in treatment of different tumor types.

## Cancer therapy and angiogenesis

### The process of tumor angiogenesis

In recent years, different treatment strategies have been proposed for the treatment of cancers. Generation of new blood vessels (neoangiogenesis) from pre-existing vasculature during the embryogenesis is characterized as a fundamental process that leads to development of concomitant vascular network [65]. Neoangiogenesis in common physiological sate is considered a stable event that are rarely observed in adults. That notwithstanding, angiogenesis might be occurred in adults during pregnancy as well as the ovarian corpus luteum development [66]. In addition, neoangiogenesis is a critical key event in pathological conditions, such as tumor progression [67]. However, there is unique characteristics of tumor cells vessels compared with normal vessels with respect to architecture and structure [68]. Tumor vasculature commonly lacks organization and is and mazy, and usually has no organized layers of venules, arterioles, and capillaries. With regard to molecular structure, ECs of tumor tissues have been reported to highly express Placental growth factor (PlGF), CD137, CD109, and CD276. That notwithstanding, the origin of ECs in tumor tissues has not been clearly revealed [69]. However, studies have established that endothelial progenitor cells (EPCs) are primarily involved in the angiogenesis of tumor tissue [69]. An imbalance between factors triggering and suppression angiogenesis in tumor microenvironment is required for initiation of angiogenesis. The most key triggering factors of angiogenesis in a tumor tissue include MMPs, vascular endothelial growth factor-A (VEGF-A), fibroblast growth factor (FGF), hepatocyte growth factor (HGF), and PlGF, [70, 71]. Conversely, the angiogenetic suppressors include thrombospondins (THSBs), IL-12, endostatin, and angiostatin [72].

### Anti-angiogenesis tumor therapy through inhibition of VEGF

Considering the participation of angiogenesis and vascularization the development and progression of cancers, it seems that inhibition of tumor angiogenesis confers a therapeutic strategy. Basically, inhibition of growth factors/signalling pathways necessary for progression and growth of ECs is regarded as a vital approach to supers the tumor angiogenesis [67, 73]. It has been shown that vascular endothelial growth factor (VEGF) plays a key modulatory function during the process of angiogenesis not only in the physiological conditions, but also in the pathological settings [74]. Moreover, it was reported that VEGF-null embryonic stem cells could not develop construct teratoma in a recipient after inoculation in testis capsule, suggesting that VEGF and angiogenesis are fundamentally involved in the tumor growth and development. Hypoxic conditions in different areas of cancers cause upregulation of VEGF in the tumor microenvironment, implying to the expedited growth of cancer cells and defective blood flow [75].

It was shown that treatment of mice with human tumor by an anti-VEGF neutralizing monoclonal antibody (mAb) resulted in fundamental repression of the tumor expansion in the animal [76]. Bevacizumab, a humanized variant of a VEGF neutralizing mAb [77], was approved by the Food & Drug Administration (FDA) as the first anti-angiogenic treatment for therapy with standard of care (SOC) in subjects suffering from metastatic colorectal cancer (CRC) [78]. Afterwards, bevacizumab was then approved to have positive therapeutic effects in individuals with non-small-cell lung cancer (NSCLC) [79] and metastatic breast cancer (BC) [80].

Upon successful experiences, several agents inhibiting the VEGF pathway have been developed that are in the clinical trial evaluations, which target either VEGF or the related receptor. AS a chimeric soluble receptor of VEGF, VEGF-TrapR1R2 contain functional sections of VEGFR1 and VEGFR2 [81], and thereby, cab attach to and neutralize circulating VEGF molecules. Additionally, clinical studies on animal models have reported that VEGF-TrapR1R2, in comparison to other VEGF receptor blockers such as DC101, may have better anti-tumor function.

Inhibition of VEGF signalling pathway by blocking the VEGFR has been accompanied with promising outcomes [82, 83]. For example, receptor tyrosine kinase inhibitors (RTKIs) like linifanib [84, 85], cabozantinib [86], axitinib [87], tivozatinib [88], vendatanib [89], sunitinib [90], pazopanib [91], and sorafenib [92] have been assessed. Although the clinical studies establish the positive effects of these agents on tumor repression, the exact mechanism of action of them has not been divulged yet. Studies have indicated that angiogenesis inhibitors against VEGF pathway suppress tumor development by interfering the angiogenesis of tumor tissue. That notwithstanding, these agents may act by vascular normalization, through which they function on the non-functional vessels in tumors, resulting in increased blood flow and, thereby, improved delivery of cytotoxic agents to the tumor tissue for the aim of killing tumor cells [93].

Conflicting outcomes have been approached with respect to the efficacy of VEGF inhibitors between preclinical models and clinical trials. Through clinical trials, it has been observed that the efficacy of mAbs might be different compared with the small anti-angiogenesis blockers. Studies have revealed that bevacizumab combined with SOC had suitable results in individuals with metastatic BC [80], CRC [78], and NSCLC [79]. In general, development of VEGF blockers as well as anti-angiogenic agents have conferred a promising therapeutic tool for tumors.

Besides VEGF blockers, vascular disrupting agents (VDAs) have also been developed to inhibit angiogenesis. VDAs action in inhibition of the tumor development is through stimulation vascular collapse, leading to hypoxia and, therefore, cancer cells necrosis [94]. As a VDA, ASA404 is a flavonoid factor and stimulates apoptosis in tumor ECs, resulting in interruption of blood flow in tumor tissues. Nowadays, ASA404 is under clinical trials in NSCLC patients [95] (Table 2). Application of vascular VDAs might be promising in repressing tumor angiogenesis, but further investigations is still needed to ensure their efficacy in the clinical use during different tumor types.

### Anti-angiogenesis tumor therapy through inhibition of receptor tyrosine kinases

Ligation of growth factors to their corresponding receptors leads to acceleration of the process of angiogenesis, resulting in activation and signal transduction through receptor tyrosine kinases (RTKs) [96]. A number of small molecules that are involved in the prevention of the angiogenesis disturbs the phosphorylation of VEGFR-2, and thereby inhibit the expansion of ECs and development of blood vessel cells [97]. As a potential therapeutic approach, RTKs can be targeted through devising novel anti-angiogenesis factors. In this way, sorafenib, as a kinase inhibitor, are involved in prevention of tumor growth through interrupting in proliferation and antiangiogenic effects. Additionally, sorafenib has been shown to possess antitumor function according to the phase III trials in patients with hepatocellular carcinoma as well as advanced renal cell carcinoma [98]. Furthermore, sunitinib in a phase III clinical trial in gastrointestinal stromal tumor patients was accompanied with promising outcomes [99] (Table 2). Although inhibitors of RTKs have been associated with remarkable outcomes in different cancers, their general response rate is not considerably satisfying. Additionally, such agents might be toxic and accompany with resistance against the anti-angiogenic targeted agents.

## MSCs-based cancer therapy by inhibition of angiogenesis

**Fig. 3:**
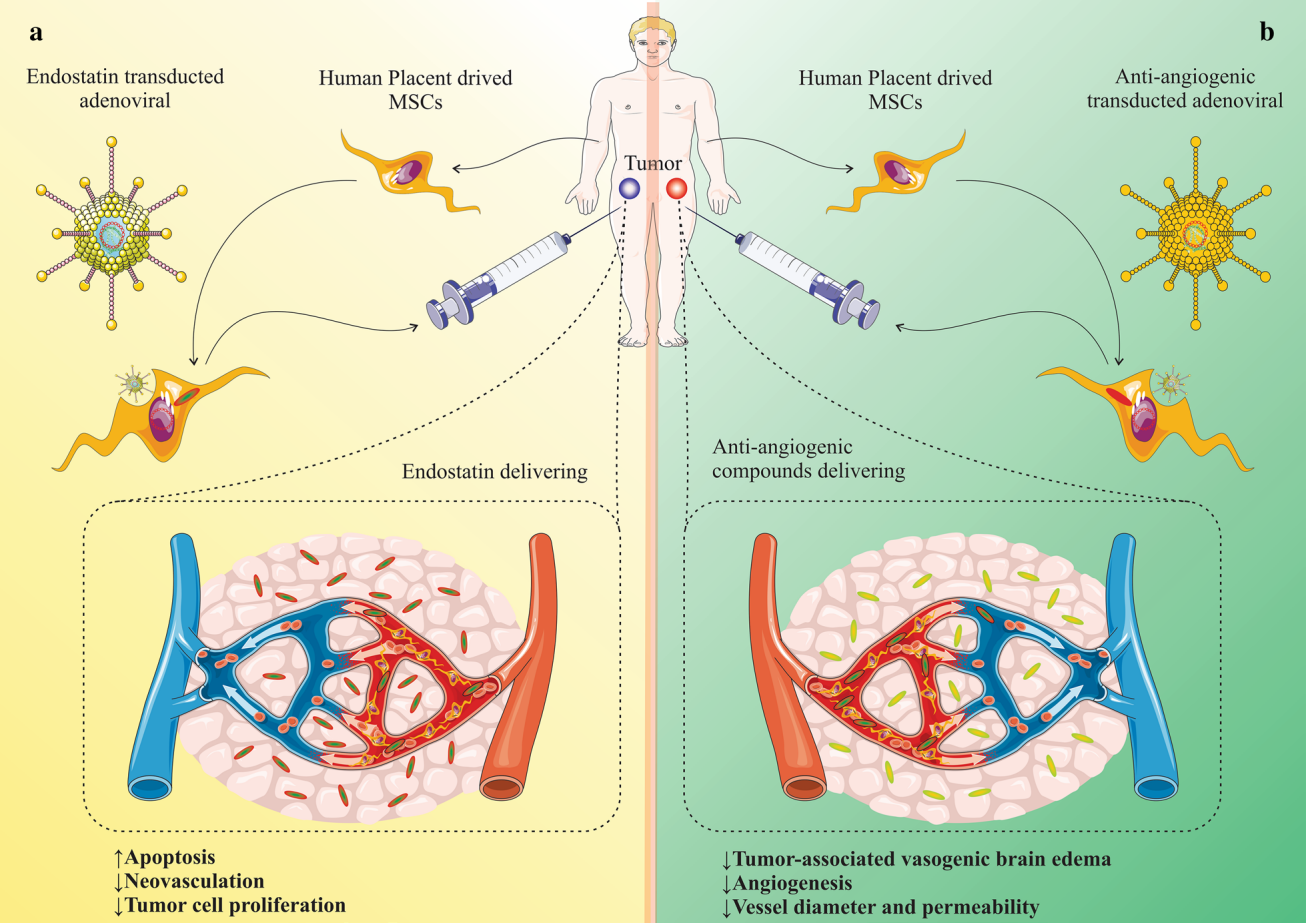
**a** Adenoviral transduction is employed to modulate the human placenta-derived-MSCs for delivering endostatin. Such MSCs expressing human endostatin are homed into the cancer tissue and remarkably mitigate the size of tumor without considerable systemic toxic adverse effects. The beneficial effects in favour of tumor treatment are due to the promoted apoptosis of cancer cell as well as disturbed neovascularization, thereby decreased tumor cell proliferation and decelerated expansion. **b** Delivering the anti-angiogenic compounds resulted in normalization of the aberrant constructions and impaired function of the blood vessels, resulting in a remarkable diminished tumor-associated vasogenic brain edema, decreased vessel diameter and permeability was the underlying cause of the vessel normalization

Angiogenesis is a key event involved in the perpetuation of the growth and progression of tumor cells [9]. Employing MSC vehicles can be potentially considered as one of the approaches to deliver anti-angiogenetic compounds to confine the tumor expansion and metastasis. Such modified MSCs have been accompanied with a tropism to tumor tissue and can deliver antiangiogenic factors that lack unwanted adverse effects [100]. That notwithstanding, systemic and chronic delivering of the anti-angiogenic agents has been attributed with toxicity, and poor blood circulation, which reduces the delivering capacity of the chemotherapeutic drugs to the cancer cells [101]. Tumor-associated angiogenesis has been established to originate from an imbalance in the regulation of both antiangiogenic and pro-angiogenic factors along with through growth factors expressed in the tumor niche [10, 102]. As an remarkable endogenous inhibitor of the angiogenesis in the tumors, endostatin has been extensively exerted to interrupt the antiangiogenic for the aim of treating diverse malignancies [103]. In an animal study, adenoviral transduction was employed to modulate the human placenta-derived MSCs for delivering endostatin that were injected into nude mice. Such MSCs that expressed human endostatin homed into the cancer tissue and remarkably mitigated the size of tumor without considerable systemic toxic adverse impressions. The boon effects in favour of tumor treatment underlined the promoted apoptosis of cancer cell as well as disturbed neovascularization, thereby decreased tumor cell proliferation and decelerated expansion [103] (Fig. 3a). Furthermore, delivering the anti-angiogenic compounds in a phase II clinical trial resulted in normalization of the abnormal constructions and impaired function of the blood vessels, resulting in a remarkable diminished tumor-associated vasogenic brain edema [104]. Decreased vessel diameter and permeability was the underlying cause of the vessel normalization [105, 106]. As such, promoted mural cell coating of the small vessels was another contributing issue for vessel normalization [107]. Investigations demonstrate that MSCs have the potential in localization to tumor vasculature following intratumoral injection, proposing boon features for targeted delivery of anti-angiogenic compounds, particularly in vascularized malignancies [108] (Fig. 3b).

Exosomes derived from MSCs are able to deliver miRs into the target cells with the aim of modulation of angiogenesis in tumors. Exposure of MSCs with the exosome secretion blocker GW4869 led to reduced levels of pro-angiomiRs in the MSC-derived medium [109]. These observations propose that exosomal transfer of pro-angiogenic miRs are vitally involved in MSC-associated angiogenesis and the communication between MSC and endothelial cell [109]. An anti-tumor role of intra-tumoral injection of MSC-derived exosomes was led to inhibition of angiogenesis in hamster buccal pouch carcinoma as a preclinical model for human oral squamous cell carcinoma [110]. It was also observed that exosome-treatment of endothelial cells resulted in promoted cytotoxicity and downmodulation of VEGF secretion and angiogenesis [110]. Other study reported that after MSCs injection, corneal neovascularization was decreased in human umbilical vein endothelial cells (HUVECs). These MSCs prevented the angiogenesis of HUVECs by upmodulating miR-211, which targeted and suppressed the expression of prospero homeobox 1 (PROX1) [111]. Other research demonstrated that MSC-derived exosomes, containing miR-16 (that target and suppress VEGF), could downmodulate the expression of VEGF in tumor cells, culminating in in vitro and in vivo suppression of angiogenesis [112]. MSC-derived exosomes may play a significant role in mediating the cell-to-cell cross-talk in the tumor microenvironment and are able to repress the angiogenesis through transferring anti-angiogenic molecules. As a result, targeted delivering of exosomes expressing miRNAs to inhibit angiogenetic molecules using MSCs may provide a potential approach in hindering angiogenesis and tumor development.

The inflammatory setting of tumor microenvironment induces the local recruitment of MSCs. Pro-inflammatory mediators produced in the tumor microenvironment might influence on the phenotype and functional characteristics of MSCs that finally results in MSC-mediated angiogenesis of tumor. In vitro investigations demonstrated that tumor necrosis factor (TNF)-α, as an inflammatory cytokine, was able to stimulate the expression of VEGF in the intra-pancreatic tissue-derived (IPTD) MSCs. Moreover, the secreted VEGF was able to enhance angiogenesis in the human endothelial cells [113]. Yang et al. indicated that pro-inflammatory cytokines, such as TNF-α and interferon (IFN-γ) triggered MSCs to overexpress platelet-derived growth factor (PDGF) and VEGF, therefore promoted angiogenic effects of MSCs on the prostate cancer in mice [114]. Additionally, both IFN-γ and TNF-α were observed to stimulate MSCs to produce higher levels of VEGF in the tumor microenvironment, leading to enhanced angiogenesis in colon cancer of mice [115]. Wang et al. reported that MSC-derived IL-8 (rather that CRC-derived IL-8) had a substantial effect on the pro-angiogenic properties of MSCs. In fact, MSC-derived IL-8 was able to induce angiogenesis through proliferation of HUVECs [116]. Moreover, MSC-derived IL-6 was shown to promote the secretion of endothelin-1 (ET-1) in the CRC cancer cells, leading to activation of Akt and ERK in the endothelial cells, hence upmodulate the angiogenesis of CRC cells [117]. All these data support the involvement of the inflammatory mediators in promoting the pro-angiogenic effects of MSCs.

## Clinical trials on cancer therapy underlying MSC application

Over the course of past decade, there has been a quick progression on the application of cells in treatment of cancers, and MSCs-based therapies has been on the fast track even further. Despite the general anti-tumor characteristics of MSCs, these cells conferred a potential for the personalized cell-based treatments due to the ease of availability with little invasive implementations on the individuals along with a great potential of expansion in vitro [118]. At the moment, according to the ClinicalTrials.gov records, there are 25 registered trials intending to utilize MSCs through diverse approaches for amelioration of tumors. The majority of such clinical trials are currently under implementation in the phase I or II that aimed to assess the adverse effects, safety and efficacy of MSC therapy in the individuals under study.

The 2013 phase I/II clinical trial conducted in the Hospital Universitario Niño Jesús, Madrid, Spain, evaluated the application of bone marrow-derived autologous MSCs that were transduced with the oncolytic adenovirus Celyvir for treating refractory and metastatic solid tumors both in adults and children [119]. In this trial, 20 subjects received weekly (n = 6) intravenous infusion of Celyvir. This study assessed the side effects of infusions after 48 h and the clinical observations were monitored for up to 2 months after the last infusion. The trial indicated that multidoses of Celyvir had an acceptable safety as well as prosperous outcomes in treating the tumor [120, 121].

The Treatment of Advanced Gastrointestinal Tumors with Genetically Modified Autologous Mesenchymal Stromal Cells (TREAT-ME1) phase I/II clinical trial delivered herpes simplex virus thymidine kinase (HSV-TK) by genetically modified autologous MSCs as a delivery vehicle and monitored the safety and efficacy of the treatment [122]. A phase I clinical trial by The University of Texas M.D. Anderson Cancer Center applied the human IFN-β-transduced MSCs to determine the side effects, safety and the well-tolerable dose in patients with ovarian cancer. Additionally, the Mayo Clinic performed a phase I/II trial in order to identify the side effects as well as the optimal dose of MSCs transfected with oncolytic measles virus encoding NIS (MV-NIS) in the ovarian cancer. A phase I clinical trial in prostate cancer subjects, investigated the potential of allogeneic bone-marrow MSCs for homing in the target sites. The trial reported that MSCs were not able to home in tumor sites efficiently to modulate desired therapeutic functions [123].

Among the clinical trials, nine studies assessed the potential of MSCs in the amelioration of adverse outcomes during the conventional anti-cancer therapies, for example radiation-induced hemorrhagic cystitis (NCT02814864), radiotherapy-induced xerostomia (NCT03874572), anthracyclines-induced cardiomyopathy (NCT02509156), cisplatin-induced acute renal failure (NCT01275612). As a result, MSC-associated cell-based cancer therapy confer a therapeutic approach in the treatment of side effects by other anti-cancer therapies, along with direct application of MSCs in cancer therapy. Although a promising progression have been made with respect to exploring the potential of MSCs in cancer therapy, this therapeutic system is in its infancy and it will take a great deal of work to transform the preclinical and clinical findings into real clinical practice (Table 3).

**Table 3:** Clinical trials aiming to cancer treatment using MSCs

Cancer type	Trial NCT number	Treatment approach	Phase	Country	Status	Year
Hematological malignancies	NCT01045382	MSC	II	Belgium	Recruiting	2010
Hematological malignancies	NCT01092026	Umbilical cord blood hematopoietic stem cell (UCB-HSC) transplantation with co-infusion of MSCs	I, II	Belgium	Unknown	2010
Myelodysplastic syndromes	NCT01129739	MSCs derived from human umbilical cord/placenta	II	China	Unknown	2010
Solid tumors metastases	NCT01844661	Bone marrow-derived autologous MSCs infected with ICOVIR5 (Celyvir)	I, II	Spain	Completed	2013
Prostate cancer	NCT01983709	Bone marrow-derived MSCs	I	US	Terminated (Study was Terminated by PI due to low accrual)	2013
Hematological malignancies	NCT02270307	Cyclophosphamide and MSC	II, III	Russia	Unknown	2014
Ovarian cancer	NCT02068794	Tissue-derived MSCs infected with oncolytic measles virus encoding thyroidal sodium iodide symporter (AdMSC-MV-NIS)	I, II	US	Recruiting	2014
Hematological malignancies	NCT02181478	Intra-osseous umbilical cord blood hematopoietic stem cells (UC-HSC) and MSC	I	US	Completed	2015
Ovarian cancer	NCT02530047	Bone marrow-derived MSCs expressing INF-β (BM-MSC-β)	I	US	Completed	2016
Myelodysplastic syndromes	NCT03184935	Human umbilical cord-derived MSCs	I, II	China	Suspended	2017
Lung adenocarcinoma	NCT03298763	MSCs genetically engineered to express TRAIL (MSC-TRAIL)	I, II	UK	Recruiting	2019
Pancreatic cancer	NCT03608631	MSC-derived exosomes loaded with KrasG12D siRNA	I	US	Not yet recruiting	2019
Glioma	NCT03896568	Allogeneic bone marrow-derived MSCs loaded with the oncolytic adenovirus DNX-2401 (BM-MSCs-DNX2401	I	US	Recruiting	2019

## Challenges and limitations of MSC-based cancer therapy

Although application of modified MSCs has conferred a novel and promising treatment option in cancer therapy, a number of challenging issues have been raised that might limit their use. As an instance, proper homing of MSCs in the target sites as well as their survival after engraftment are potential limitations. Biomaterial scaffolds may promote the suitable engraftment of the delivered MSCs in the target regions [124–126]. For treatment of postoperative brain cancer, researchers developed an approach to deliver the engendered MSCs on the biomaterials [127]. In this method, MSCs are seeded on the biodegradable fibrin scaffolds that are implanted into the region under surgery to combat the residual cancer cells, leading to promoting the potential of tumor-free survival. In acute myeloid leukemia, researchers applied cryogel-housed MSCs modulated to secrete a bispecific (anti-CD3 and anti-CD33) antibody for maximizing cancer immunotherapy [128]. Biomaterials may also be utilized to encapsulate the MSCs, resulting in protective ability within the host’s body along with conferring the potential of releasing drugs and providing an MSC-nourishing microenvironment. Cellulose, alginate and agarose have shown favourable results in designing biomaterial microcapsules for MSCs [129–131]. It was reported that alginate-encapsulated cells in the glioblastoma patients had beneficial effects in preventing cancer growth [131]. As a consequence, use of biomaterials in the engineered MSCs systems for cancer therapy possess the potential to deter the limitations and improve the treatment efficacy, but needs further researches.

## Conclusions and future directions

MSCs can preferentially migrate into the tumor tissues and have transactions with various cells in the tumor microenvironment. These cells are easily accessible, do not induce immunological responses, can be simply manipulated in vitro without requirement for immortalization; therefore, they are the most suitable choices for cell-based treatments in cancers. In spite of progress in obtaining sufficient amount of autologous and allogeneic MSCs from bone marrow, adipose tissue, umbilical cord blood, and local tissues, nonetheless, their regulation and kinetics requires further elucidation. Moreover, engineering the exosomes released by MSCs may offer another promising technique in MSC-based cancer therapy.

In spite of providing novel and attracting therapeutic system in cancer therapy, MSCs has been accompanied by a number of challenging issues and limitations. Efficient homing of MSCs in the target regions and their survival after engraftment seem to be challenges in the way toward MSC-based cancer therapy. Nonetheless, a number of recent studies have tried to utilize the biomaterials to come up with solutions for such seatbacks. It should be noted that we just started to understand the behaviours of MSCs and exert them in the clinical trials. We need to be armed with identification of new hypotheses in exertion of anti-angiogenic agents that have little adverse effects. Reaching to an optimal anti-angiogenic treatment strategy requires elaborated solutions. Manipulation of MSCs for precise delivering of the angiogenesis inhibitors could be still the first option. That notwithstanding, searching for novel molecular targets as well as agents with less side effects may help to improve the anti-angiogenesis therapy of tumors in the future.

## Data Availability

The datasets used and/or analysed during the current study available from the corresponding author on reasonable request.

## Abbreviations

PTX: Paclitaxel
GO: Graphene oxide
hAFMSCs: Human amniotic fluid mesenchymal stem cells
TRAIL: Tumor necrosis factor-related apoptosis-inducing ligand
HDACi: Different histone deacetylase inhibitors
VPA: Valproic acid
SAHA: Suberanilohydroxamic acid
EPCs: Endothelial progenitor cells
MMPs: Matrix metalloproteinases
VEGF-A: Vascular endothelial growth factor-A
FGF: Fibroblast growth factor
HIF-1a: Hypoxia inducible factor
mAb: Monoclonal antibody
FDA: Food & Drug Administration
SOC: Standard of care
CRC: Colorectal cancer
NSCLC: Non-small-cell lung cancer
BC: Breast cancer
RTKIs: Receptor tyrosine kinase inhibitors
VDAs: Vascular disrupting agents
RTKs: Receptor tyrosine kinases
AngGF: Angiogenic growth factor

## References

[1] Afkham A, Aghebati-Maleki L, Siahmansouri H, Sadreddini S, Ahmadi M, Dolati S, Afkham NM, Akbarzadeh P, Jadidi-Niaragh F, Younesi V (2018). Chitosan (CMD)-mediated co-delivery of SN38 and Snail-specific siRNA as a useful anticancer approach against prostate cancer. Pharmacol Rep.

[2] Aghebati-Maleki A, Dolati S, Ahmadi M, Baghbanzhadeh A, Asadi M, Fotouhi A, Yousefi M, Aghebati-Maleki L (2020). Nanoparticles and cancer therapy: perspectives for application of nanoparticles in the treatment of cancers. J Cell Physiol.

[3] Siahmansouri H, Somi MH, Babaloo Z, Baradaran B, Jadidi-Niaragh F, Atyabi F, Mohammadi H, Ahmadi M, Yousefi M (2016). Effects of HMGA 2 si RNA and doxorubicin dual delivery by chitosan nanoparticles on cytotoxicity and gene expression of HT-29 colorectal cancer cell line. J Pharm Pharmacol.

[4] Shali H, Ahmadi M, Kafil HS, Dorosti A, Yousefi M (2016). IGF1R and c-met as therapeutic targets for colorectal cancer. Biomed Pharmacother.

[5] Sawyers C (2004). Targeted cancer therapy. Nature.

[6] Collaboration GBoDC (2019). Global, regional, and national cancer incidence, mortality, years of life lost, years lived with disability, and disability-adjusted life-years for 29 cancer groups, 1990 to 2017: a systematic analysis for the global burden of disease study. JAMA oncol.

[7] Teo AK, Vallier L (2010). Emerging use of stem cells in regenerative medicine. Biochem J.

[8] Wei X, Yang X, Han Z-P, Qu F-F, Shao L, Shi Y-F (2013). Mesenchymal stem cells: a new trend for cell therapy. Acta Pharmacol Sin.

[9] Jain RK, Di Tomaso E, Duda DG, Loeffler JS, Sorensen AG, Batchelor TT (2007). Angiogenesis in brain tumours. Nat Rev Neurosci.

[10] Samant RS, Shevde LA (2011). Recent advances in anti-angiogenic therapy of cancer. Oncotarget.

[11] Friedenstein A, Chailakhyan R, Gerasimov U (1987). Bone marrow osteogenic stem cells: in vitro cultivation and transplantation in diffusion chambers. Cell Prolif.

[12] Aghebati‐Maleki L, Dolati S, Zandi R, Fotouhi A, Ahmadi M, Aghebati A (2019). Prospect of mesenchymal stem cells in therapy of osteoporosis: a review. Biosci Rep.

[13] Dominici M, Le Blanc K, Mueller I, Slaper-Cortenbach I, Marini F, Krause D, Deans R, Keating A, Prockop D, Horwitz E (2006). Minimal criteria for defining multipotent mesenchymal stromal cells. The International Society for Cellular Therapy position statement. Cytotherapy.

[14] Devine SM, Hoffman R (2000). Role of mesenchymal stem cells in hematopoietic stem cell transplantation. Curr Opin Hematol.

[15] Shi Y, Du L, Lin L, Wang Y (2017). Tumour-associated mesenchymal stem/stromal cells: emerging therapeutic targets. Nat Rev Drug Discov.

[16] He Q, Wan C, Li G (2007). Concise review: multipotent mesenchymal stromal cells in blood. Stem cells.

[17] Hong HS, Lee J, Lee E, Kwon YS, Lee E, Ahn W, Jiang MH, Kim JC, Son Y (2009). A new role of substance P as an injury-inducible messenger for mobilization of CD29+ stromal-like cells. Nat Med.

[18] Momin NE, Vela G, Zaidi AH, Quiñones-Hinojosa A (2010). The oncogenic potential of mesenchymal stem cells in the treatment of cancer: directions for future research. Curr Immunol Rev.

[19] Papait A, Stefani FR, Cargnoni A, Magatti M, Parolini O, Silini AR (2020). The multifaceted roles of MSCs in the tumor microenvironment: interactions with immune cells and exploitation for therapy. Front Cell Dev Biol.

[20] Patel SA, Meyer JR, Greco SJ, Corcoran KE, Bryan M, Rameshwar P (2010). Mesenchymal stem cells protect breast cancer cells through regulatory T cells: role of mesenchymal stem cell-derived TGF-β. J Immunol.

[21] Eghbal-Fard S, Yousefi M, Heydarlou H, Ahmadi M, Taghavi S, Movasaghpour A, Jadidi-Niaragh F, Yousefi B, Dolati S, Hojjat-Farsangi M, Rikhtegar R, Nouri M, Aghebati-Maleki L (2019). The imbalance of Th17/Treg axis involved in the pathogenesis of preeclampsia. J Cell Physiol.

[22] Waterman RS, Tomchuck SL, Henkle SL, Betancourt AM (2010). A new mesenchymal stem cell (MSC) paradigm: polarization into a pro-inflammatory MSC1 or an Immunosuppressive MSC2 phenotype. PLoS ONE.

[23] Zhu Y, Sun Z, Han Q, Liao L, Wang J, Bian C, Li J, Yan X, Liu Y, Shao C (2009). Human mesenchymal stem cells inhibit cancer cell proliferation by secreting DKK-1. Leukemia.

[24] Lu Y-R, Yuan Y, Wang X-J, Wei L-L, Chen Y-N, Cong C, Li S-F, Long D, Tan W-D, Mao Y-Q (2008). The growth inhibitory effect of mesenchymal stem cells on tumor cells in vitro and in vivo. Cancer Biol Ther.

[25] Qiao L, Xu Z-L, Zhao T-J, Ye L-H, Zhang X-D (2008). Dkk-1 secreted by mesenchymal stem cells inhibits growth of breast cancer cells via depression of Wnt signalling. Cancer Lett.

[26] Otsu K, Das S, Houser SD, Quadri SK, Bhattacharya S, Bhattacharya J (2009). Concentration-dependent inhibition of angiogenesis by mesenchymal stem cells. Blood.

[27] Suzuki K, Sun R, Origuchi M, Kanehira M, Takahata T, Itoh J, Umezawa A, Kijima H, Fukuda S, Saijo Y (2011). Mesenchymal stromal cells promote tumor growth through the enhancement of neovascularization. Mol Med.

[28] Nishimura K, Semba S, Aoyagi K, Sasaki H, Yokozaki H (2012). Mesenchymal stem cells provide an advantageous tumor microenvironment for the restoration of cancer stem cells. Pathobiology.

[29] Liu S, Ginestier C, Ou SJ, Clouthier SG, Patel SH, Monville F, Korkaya H, Heath A, Dutcher J, Kleer CG (2011). Breast cancer stem cells are regulated by mesenchymal stem cells through cytokine networks. Can Res.

[30] Corcoran KE, Trzaska KA, Fernandes H, Bryan M, Taborga M, Srinivas V, Packman K, Patel PS, Rameshwar P (2008). Mesenchymal stem cells in early entry of breast cancer into bone marrow. PLoS ONE.

[31] Karnoub AE, Dash AB, Vo AP, Sullivan A, Brooks MW, Bell GW, Richardson AL, Polyak K, Tubo R, Weinberg RA (2007). Mesenchymal stem cells within tumour stroma promote breast cancer metastasis. Nature.

[32] Djouad F, Plence P, Bony C, Tropel P, Apparailly F, Sany J, Noël D, Jorgensen C (2003). Immunosuppressive effect of mesenchymal stem cells favors tumor growth in allogeneic animals. Blood.

[33] Ren G, Zhao X, Wang Y, Zhang X, Chen X, Xu C, Yuan Z-R, Roberts AI, Zhang L, Zheng B (2012). CCR2-dependent recruitment of macrophages by tumor-educated mesenchymal stromal cells promotes tumor development and is mimicked by TNFα. Cell Stem Cell.

[34] Razmkhah M, Jaberipour M, Erfani N, Habibagahi M, Talei A-R, Ghaderi A (2011). Adipose derived stem cells (ASCs) isolated from breast cancer tissue express IL-4, IL-10 and TGF-β1 and upregulate expression of regulatory molecules on T cells: do they protect breast cancer cells from the immune response?. Cell Immunol.

[35] McLean K, Gong Y, Choi Y, Deng N, Yang K, Bai S, Cabrera L, Keller E, McCauley L, Cho KR (2011). Human ovarian carcinoma–associated mesenchymal stem cells regulate cancer stem cells and tumorigenesis via altered BMP production. J Clin Investig.

[36] Le Page C, Puiffe M-L, Meunier L, Zietarska M, de Ladurantaye M, Tonin PN, Provencher D, Mes-Masson A-M (2009). BMP-2 signaling in ovarian cancer and its association with poor prognosis. J Ovar Res.

[37] Xu W-T, Bian Z-Y, Fan Q-M, Li G, Tang T-T (2009). Human mesenchymal stem cells (hMSCs) target osteosarcoma and promote its growth and pulmonary metastasis. Cancer Lett.

[38] Spaeth EL, Dembinski JL, Sasser AK, Watson K, Klopp A, Hall B, Andreeff M, Marini F (2009). Mesenchymal stem cell transition to tumor-associated fibroblasts contributes to fibrovascular network expansion and tumor progression. PLoS ONE.

[39] Zhu W, Xu W, Jiang R, Qian H, Chen M, Hu J, Cao W, Han C, Chen Y (2006). Mesenchymal stem cells derived from bone marrow favor tumor cell growth in vivo. Exp Mol Pathol.

[40] Okumura T, Wang SS, Takaishi S, Tu SP, Ng V, Ericksen RE, Rustgi AK, Wang TC (2009). Identification of a bone marrow-derived mesenchymal progenitor cell subset that can contribute to the gastric epithelium. Lab Invest.

[41] Raposo G, Stoorvogel W (2013). Extracellular vesicles: exosomes, microvesicles, and friends. J Cell Biol.

[42] Braicu C, Tomuleasa C, Monroig P, Cucuianu A, Berindan-Neagoe I, Calin G (2015). Exosomes as divine messengers: are they the Hermes of modern molecular oncology?. Cell Death Differ.

[43] Zhang L, Hao C, Yao S, Tang R, Guo W, Cong H, Li J, Bao L, Wang D, Li Y (2018). Exosomal miRNA profiling to identify nanoparticle phagocytic mechanisms. Small.

[44] Guescini M, Genedani S, Stocchi V, Agnati LF (2010). Astrocytes and Glioblastoma cells release exosomes carrying mtDNA. J Neural Trans.

[45] Neviani P, Fabbri M (2015). Exosomic microRNAs in the tumor microenvironment. Front Med.

[46] Ratajczak J, Miekus K, Kucia M, Zhang J, Reca R, Dvorak P, Ratajczak M (2006). Embryonic stem cell-derived microvesicles reprogram hematopoietic progenitors: evidence for horizontal transfer of mRNA and protein delivery. Leukemia.

[47] Kucerova L, Altanerova V, Matuskova M, Tyciakova S, Altaner C (2007). Adipose tissue–derived human mesenchymal stem cells mediated prodrug cancer gene therapy. Can Res.

[48] Cihova M, Altanerova V, Altaner C (2011). Stem cell based cancer gene therapy. Mol Pharm.

[49] Matuskova M, Hlubinova K, Pastorakova A, Hunakova L, Altanerova V, Altaner C, Kucerova L (2010). HSV-tk expressing mesenchymal stem cells exert bystander effect on human glioblastoma cells. Cancer Lett.

[50] Altanerova U, Jakubechova J, Benejova K, Priscakova P, Pesta M, Pitule P, Topolcan O, Kausitz J, Zduriencikova M, Repiska V (2019). Prodrug suicide gene therapy for cancer targeted intracellular by mesenchymal stem cell exosomes. Int J Cancer.

[51] Rowinsky EK, Donehower RC (1995). Paclitaxel (taxol). N Engl J Med.

[52] Kalimuthu S, Gangadaran P, Rajendran RL, Zhu L, Oh JM, Lee HW, Gopal A, Baek SH, Jeong SY, Lee S-W (2018). A new approach for loading anticancer drugs into mesenchymal stem cell-derived exosome mimetics for cancer therapy. Front Pharmacol.

[53] Bagheri E, Abnous K, Farzad SA, Taghdisi SM, Ramezani M, Alibolandi M (2020). Targeted doxorubicin-loaded mesenchymal stem cells-derived exosomes as a versatile platform for fighting against colorectal cancer. Life Sci.

[54] Xunian Z, Kalluri R (2020). Biology and therapeutic potential of mesenchymal stem cell-derived exosomes. Cancer Sci.

[55] Goenka S, Sant V, Sant S (2014). Graphene-based nanomaterials for drug delivery and tissue engineering. J Control Release.

[56] Kang NH, Hwang KA, Yi BR, Lee HJ, Jeung EB, Kim SU, Choi KC (2012). Human amniotic fluid-derived stem cells expressing cytosine deaminase and thymidine kinase inhibits the growth of breast cancer cells in cellular and xenograft mouse models. Cancer Gene Ther.

[57] Gholizadeh-Ghaleh Aziz S, Fardyazar Z, Pashaiasl M (2019). The human amniotic fluid mesenchymal stem cells therapy on, SKOV3, ovarian cancer cell line. Mol Genet Genomic Med.

[58] Kasuga C, Ebata T, Kayagaki N, Yagita H, Hishii M, Arai H, Sato K, Okumura K (2004). Sensitization of human glioblastomas to tumor necrosis factor-related apoptosis-inducing ligand (TRAIL) by NF-kappaB inhibitors. Cancer Sci.

[59] Sanlioglu AD, Dirice E, Aydin C, Erin N, Koksoy S, Sanlioglu S (2005). Surface TRAIL decoy receptor-4 expression is correlated with TRAIL resistance in MCF7 breast cancer cells. BMC Cancer.

[60] Cornago M, Garcia-Alberich C, Blasco-Angulo N, Vall-Llaura N, Nager M, Herreros J, Comella JX, Sanchis D, Llovera M (2014). Histone deacetylase inhibitors promote glioma cell death by G2 checkpoint abrogation leading to mitotic catastrophe. Cell Death Dis.

[61] Schuler S, Fritsche P, Diersch S, Arlt A, Schmid RM, Saur D, Schneider G (2010). HDAC2 attenuates TRAIL-induced apoptosis of pancreatic cancer cells. Mol Cancer.

[62] Choi SA, Lee YE, Kwak PA, Lee JY, Kim SS, Lee SJ, Phi JH, Wang KC, Song J, Song SH (2015). Clinically applicable human adipose tissue-derived mesenchymal stem cells delivering therapeutic genes to brainstem gliomas. Cancer Gene Ther.

[63] Choi SA, Lee C, Kwak PA, Park CK, Wang KC, Phi JH, Lee JY, Chong S, Kim SK (2019). Histone deacetylase inhibitor panobinostat potentiates the anti-cancer effects of mesenchymal stem cell-based sTRAIL gene therapy against malignant glioma. Cancer Lett.

[64] Layek B, Sadhukha T, Panyam J, Prabha S (2018). Nano-Engineered mesenchymal stem cells increase therapeutic efficacy of anticancer drug through true active tumor targeting. Mol Cancer Ther.

[65] Wilting J, Christ B (1996). Embryonic angiogenesis: a review. Naturwissenschaften.

[66] Risau W, Flamme I (1995). Vasculogenesis. Annu Rev Cell Dev Biol.

[67] Folkman J (1971). Tumor angiogenesis: therapeutic implications. N Engl J Med.

[68] Hiratsuka S (2010). Vasculogenensis, angiogenesis and special features of tumor blood vessels. Front Biosci (Landmark edition).

[69] Ribatti D (2004). The involvement of endothelial progenitor cells in tumor angiogenesis. J Cell Mol Med.

[70] Ferrara N, Henzel WJ (1989). Pituitary follicular cells secrete a novel heparin-binding growth factor specific for vascular endothelial cells. Biochem Biophys Res Commun.

[71] Khoury CC, Ziyadeh FN (2011). Angiogenic factors. Diabetes and the Kidney.

[72] Taraboletti G, Rusnati M, Rusnati L, Ragona G (2010). Targeting tumor angiogenesis with TSP-1-based compounds: rational design of antiangiogenic mimetics of endogenous inhibitors. Oncotarget.

[73] Algire GH, Chalkley HW, Legallais FY, Park HD (1945). Vasculae reactions of normal and malignant tissues in vivo. I. vascular reactions of mice to wounds and to normal and neoplastic transplants. JNCI J Natl Cancer Inst.

[74] Ferrara N, Gerber H-P, LeCouter J (2003). The biology of VEGF and its receptors. Nat Med.

[75] Brahimi-Horn C, Pouysségur J (2006). The role of the hypoxia-inducible factor in tumor metabolism growth and invasion. Bull Cancer.

[76] Kim KJ, Li B, Winer J, Armanini M, Gillett N, Phillips HS, Ferrara N (1993). Inhibition of vascular endothelial growth factor-induced angiogenesis suppresses tumour growth in vivo. Nature.

[77] Presta LG, Chen H, O’connor SJ, Chisholm V, Meng YG, Krummen L, Winkler M, Ferrara N (1997). Humanization of an anti-vascular endothelial growth factor monoclonal antibody for the therapy of solid tumors and other disorders. Cancer Res.

[78] Hurwitz H, Fehrenbacher L, Novotny W, Cartwright T, Hainsworth J, Heim W, Berlin J, Baron A, Griffing S, Holmgren E (2004). Bevacizumab plus irinotecan, fluorouracil, and leucovorin for metastatic colorectal cancer. N Engl J Med.

[79] Sandler A, Gray R, Perry MC, Brahmer J, Schiller JH, Dowlati A, Lilenbaum R, Johnson DH (2006). Paclitaxel–carboplatin alone or with bevacizumab for non–small-cell lung cancer. N Engl J Med.

[80] Miller K, Wang M, Gralow J, Dickler M, Cobleigh M, Perez EA, Shenkier T, Cella D, Davidson NE (2007). Paclitaxel plus bevacizumab versus paclitaxel alone for metastatic breast cancer. N Engl J Med.

[81] Holash J, Davis S, Papadopoulos N, Croll SD, Ho L, Russell M, Boland P, Leidich R, Hylton D, Burova E (2002). VEGF-Trap: a VEGF blocker with potent antitumor effects. Proc Natl Acad Sci.

[82] Ellis LM, Hicklin DJ (2008). Pathways mediating resistance to vascular endothelial growth factor–targeted therapy. Clin Cancer Res.

[83] Kerbel RS (2008). Tumor angiogenesis. N Engl J Med.

[84] Jiang F, Albert DH, Luo Y, Tapang P, Zhang K, Davidsen SK, Fox GB, Lesniewski R, McKeegan EM (2011). ABT-869, a multitargeted receptor tyrosine kinase inhibitor, reduces tumor microvascularity and improves vascular wall integrity in preclinical tumor models. J Pharmacol Exp Ther.

[85] Tan E-H, Goss GD, Salgia R, Besse B, Gandara DR, Hanna NH (2011). Yang JC-H, Thertulien R, Wertheim M, Mazieres J: Phase 2 trial of Linifanib (ABT-869) in patients with advanced non-small cell lung cancer. J Thorac Oncol.

[86] You W-K, Sennino B, Williamson CW, Falcón B, Hashizume H, Yao L-C, Aftab DT, McDonald DM (2011). VEGF and c-Met blockade amplify angiogenesis inhibition in pancreatic islet cancer. Cancer Res.

[87] Ho TH, Jonasch E (2011). Axitinib in the treatment of metastatic renal cell carcinoma. Future Oncol.

[88] Eskens FA, de Jonge MJ, Bhargava P, Isoe T, Cotreau MM, Esteves B, Hayashi K, Burger H, Thomeer M, van Doorn L (2011). Biologic and clinical activity of tivozanib (AV-951, KRN-951), a selective inhibitor of VEGF receptor-1,-2, and-3 tyrosine kinases, in a 4-week-on, 2-week-off schedule in patients with advanced solid tumors. Clin Cancer Res.

[89] Langmuir P, Yver A (2012). Vandetanib for the treatment of thyroid cancer. Clin Pharmacol Ther.

[90] O'Farrell A-M, Abrams TJ, Yuen HA, Ngai TJ, Louie SG, Yee KW, Wong LM, Hong W, Lee LB, Town A (2003). SU11248 is a novel FLT3 tyrosine kinase inhibitor with potent activity in vitro and in vivo. Blood.

[91] Sleijfer S, Ray-Coquard I, Papai Z, Le Cesne A, Scurr M, Schöffski P, Collin F, Pandite L, Marreaud S, De Brauwer A (2009). Pazopanib, a multikinase angiogenesis inhibitor, in patients with relapsed or refractory advanced soft tissue sarcoma: a phase II study from the European Organisation for Research and Treatment of Cancer-Soft Tissue and Bone Sarcoma Group (EORTC study 62043). J Clin Oncol.

[92] Richly H, Henning B, Kupsch P, Passarge K, Grubert M, Hilger R, Christensen O, Brendel E, Schwartz B, Ludwig M (2006). Results of a phase I trial of sorafenib (BAY 43–9006) in combination with doxorubicin in patients with refractory solid tumors. Ann Oncol.

[93] Jain RK (2005). Normalization of tumor vasculature: an emerging concept in antiangiogenic therapy. Science.

[94] Cooney MM, van Heeckeren W, Bhakta S, Ortiz J, Remick SC (2006). Drug insight: vascular disrupting agents and angiogenesis—novel approaches for drug delivery. Nat Clin Pract Oncol.

[95] McKeage M, Von Pawel J, Reck M, Jameson M, Rosenthal M, Sullivan R, Gibbs D, Mainwaring P, Serke M, Lafitte J (2008). Randomised phase II study of ASA404 combined with carboplatin and paclitaxel in previously untreated advanced non-small cell lung cancer. Br J Cancer.

[96] Zheng X, Koh GY, Jackson T (2013). A continuous model of angiogenesis: initiation, extension, and maturation of new blood vessels modulated by vascular endothelial growth factor, angiopoietins, platelet-derived growth factor-B, and pericytes. Discrete Continuous Dyn Syst Ser B.

[97] Strawn LM, McMahon G, App H, Schreck R, Kuchler WR, Longhi MP, Hui TH, Tang C, Levitzki A, Gazit A (1996). Flk-1 as a target for tumor growth inhibition. Cancer Res.

[98] Wilhelm SM, Adnane L, Newell P, Villanueva A, Llovet JM, Lynch M (2008). Preclinical overview of sorafenib, a multikinase inhibitor that targets both Raf and VEGF and PDGF receptor tyrosine kinase signaling. Mol Cancer Ther.

[99] Le Tourneau C, Raymond E, Faivre S (2007). Sunitinib: a novel tyrosine kinase inhibitor. A brief review of its therapeutic potential in the treatment of renal carcinoma and gastrointestinal stromal tumors (GIST). Ther Clin Risk Manag.

[100] Ghaedi M, Soleimani M, Taghvaie NM, Sheikhfatollahi M, Azadmanesh K, Lotfi AS, Wu J (2011). Mesenchymal stem cells as vehicles for targeted delivery of anti-angiogenic protein to solid tumors. J Gene Med.

[101] Toi M, Hoshina S, Takayanagi T, Tominaga T (1994). Association of vascular endothelial growth factor expression with tumor angiogenesis and with early relapse in primary breast cancer. Cancer Sci.

[102] Folkman J, Watson K, Ingber D, Hanahan D (1989). Induction of angiogenesis during the transition from hyperplasia to neoplasia. Nature.

[103] Zheng L, Zhang D, Chen X, Yang L, Wei Y, Zhao X (2012). Antitumor activities of human placenta-derived mesenchymal stem cells expressing endostatin on ovarian cancer. PLoS ONE.

[104] Batchelor TT, Sorensen AG, di Tomaso E, Zhang W-T, Duda DG, Cohen KS, Kozak KR, Cahill DP, Chen P-J, Zhu M (2007). AZD2171, a pan-VEGF receptor tyrosine kinase inhibitor, normalizes tumor vasculature and alleviates edema in glioblastoma patients. Cancer Cell.

[105] Kadambi A, Carreira CM, Yun C-O, Padera TP, Dolmans DE, Carmeliet P, Fukumura D, Jain RK (2001). Vascular endothelial growth factor (VEGF)-C differentially affects tumor vascular function and leukocyte recruitment. Cancer Res.

[106] Tong RT, Boucher Y, Kozin SV, Winkler F, Hicklin DJ, Jain RK (2004). Vascular normalization by vascular endothelial growth factor receptor 2 blockade induces a pressure gradient across the vasculature and improves drug penetration in tumors. Cancer Res.

[107] Hormigo A, Gutin PH, Rafii S (2007). Tracking normalization of brain tumor vasculature by magnetic imaging and proangiogenic biomarkers. Cancer Cell.

[108] Bexell D, Gunnarsson S, Tormin A, Darabi A, Gisselsson D, Roybon L, Scheding S, Bengzon J (2009). Bone marrow multipotent mesenchymal stroma cells act as pericyte-like migratory vehicles in experimental gliomas. Mol Ther.

[109] Gong M, Yu B, Wang J, Wang Y, Liu M, Paul C, Millard RW, Xiao D-S, Ashraf M, Xu M (2017). Mesenchymal stem cells release exosomes that transfer miRNAs to endothelial cells and promote angiogenesis. Oncotarget.

[110] Rosenberger L, Ezquer M, Lillo-Vera F, Pedraza PL, Ortúzar MI, González PL, Figueroa-Valdés AI, Cuenca J, Ezquer F, Khoury M (2019). Stem cell exosomes inhibit angiogenesis and tumor growth of oral squamous cell carcinoma. Sci Rep.

[111] Pan J, Wang X, Li D, Li J, Jiang Z (2019). MSCs inhibits the angiogenesis of HUVECs through the miR-211/Prox1 pathway. J Biochem.

[112] Lee J-K, Park S-R, Jung B-K, Jeon Y-K, Lee Y-S, Kim M-K, Kim Y-G, Jang J-Y, Kim C-W (2013). Exosomes derived from mesenchymal stem cells suppress angiogenesis by down-regulating VEGF expression in breast cancer cells. PLoS ONE.

[113] Khiatah B, Qi M, Du W, Kuan T, van Megen KM, Perez RG, Isenberg JS, Kandeel F, Roep BO, Ku HT (2019). Intra-pancreatic tissue-derived mesenchymal stromal cells: a promising therapeutic potential with anti-inflammatory and pro-angiogenic profiles. Stem Cell Res Ther.

[114] Yang K-Q, Liu Y, Huang Q-H, Mo N, Zhang Q-Y, Meng Q-G, Cheng J-W (2017). Bone marrow-derived mesenchymal stem cells induced by inflammatory cytokines produce angiogenetic factors and promote prostate cancer growth. BMC Cancer.

[115] Liu Y, Han ZP, Zhang SS, Jing YY, Bu XX, Wang CY, Sun K, Jiang GC, Zhao X, Li R (2011). Effects of inflammatory factors on mesenchymal stem cells and their role in the promotion of tumor angiogenesis in colon cancer. J Biol Chem.

[116] Wang J, Wang Y, Wang S, Cai J, Shi J, Sui X, Cao Y, Huang W, Chen X, Cai Z (2015). Bone marrow-derived mesenchymal stem cell-secreted IL-8 promotes the angiogenesis and growth of colorectal cancer. Oncotarget.

[117] Huang W, Chang M, Tsai K, Hung M-C, Chen H, Hung S (2013). Mesenchymal stem cells promote growth and angiogenesis of tumors in mice. Oncogene.

[118] Escacena N, Quesada-Hernández E, Capilla-Gonzalez V, Soria B, Hmadcha A (2015). Bottlenecks in the efficient use of advanced therapy medicinal products based on mesenchymal stromal cells. Stem Cells Int.

[119] Ruano D, López-Martín JA, Moreno L, Lassaletta Á, Bautista F, Andión M, Hernández C, González-Murillo Á, Melen G, Alemany R (2020). First-in-human, first-in-child trial of autologous MSCs carrying the oncolytic virus Icovir-5 in patients with advanced tumors. Mol Ther.

[120] Garcia-Castro J, Alemany R, Cascallo M, Martinez-Quintanilla J, del Mar AM, Lassaletta A, Madero L, Ramírez M (2010). Treatment of metastatic neuroblastoma with systemic oncolytic virotherapy delivered by autologous mesenchymal stem cells: an exploratory study. Cancer Gene Ther.

[121] Melen GJ, Franco-Luzón L, Ruano D, González-Murillo Á, Alfranca A, Casco F, Lassaletta Á, Alonso M, Madero L, Alemany R (2016). Influence of carrier cells on the clinical outcome of children with neuroblastoma treated with high dose of oncolytic adenovirus delivered in mesenchymal stem cells. Cancer Lett.

[122] Niess H, von Einem JC, Thomas MN, Michl M, Angele MK, Huss R, Günther C, Nelson PJ, Bruns CJ, Heinemann V (2015). Treatment of advanced gastrointestinal tumors with genetically modified autologous mesenchymal stromal cells (TREAT-ME1): study protocol of a phase I/II clinical trial. BMC Cancer.

[123] Schweizer MT, Wang H, Bivalacqua TJ, Partin AW, Lim SJ, Chapman C, Abdallah R, Levy O, Bhowmick NA, Karp JM (2019). A phase I study to assess the safety and cancer-homing ability of allogeneic bone marrow-derived mesenchymal stem cells in men with localized prostate cancer. Stem Cells Transl Med.

[124] Zeng X, Qiu X-C, Ma Y-H, Duan J-J, Chen Y-F, Gu H-Y, Wang J-M, Ling E-A, Wu J-L, Wu W (2015). Integration of donor mesenchymal stem cell-derived neuron-like cells into host neural network after rat spinal cord transection. Biomaterials.

[125] Ql W, Hj W, Li Zh, Wang Yl Wu, Xp TY (2017). Mesenchymal stem cell-loaded cardiac patch promotes epicardial activation and repair of the infarcted myocardium. J Cell Mol Med.

[126] Diomede F, Gugliandolo A, Cardelli P, Merciaro I, Ettorre V, Traini T, Bedini R, Scionti D, Bramanti A, Nanci A (2018). Three-dimensional printed PLA scaffold and human gingival stem cell-derived extracellular vesicles: a new tool for bone defect repair. Stem Cell Res Ther.

[127] Sheets KT, Bagó JR, Hingtgen SD (2018). Delivery of cytotoxic mesenchymal stem cells with biodegradable scaffolds for treatment of postoperative brain cancer. Targeted drug delivery.

[128] Aliperta R, Welzel PB, Bergmann R, Freudenberg U, Berndt N, Feldmann A, Arndt C, Koristka S, Stanzione M, Cartellieri M (2017). Cryogel-supported stem cell factory for customized sustained release of bispecific antibodies for cancer immunotherapy. Sci Rep.

[129] Sakai S, Kawabata K, Tanaka S, Harimoto N, Hashimoto I, Mu C, Salmons B, Ijima H, Kawakami K (2005). Subsieve-size agarose capsules enclosing ifosfamide-activating cells: a strategy toward chemotherapeutic targeting to tumors. Mol Cancer Ther.

[130] Schwenter F, Zarei S, Luy P, Padrun V, Bouche N, Lee J, Mulligan R, Morel P, Mach N (2011). Cell encapsulation technology as a novel strategy for human anti-tumor immunotherapy. Cancer Gene Ther.

[131] Johansson M, Oudin A, Tiemann K, Bernard A, Golebiewska A, Keunen O, Fack F, Stieber D, Wang B, Hedman H (2013). The soluble form of the tumor suppressor Lrig1 potently inhibits in vivo glioma growth irrespective of EGF receptor status. Neuro-oncology.

[132] Uchibori R, Tsukahara T, Mizuguchi H, Saga Y, Urabe M, Mizukami H, Kume A, Ozawa K (2013). NF-κB activity regulates mesenchymal stem cell accumulation at tumor sites. Can Res.

[133] Teo GS, Ankrum JA, Martinelli R, Boetto SE, Simms K, Sciuto TE, Dvorak AM, Karp JM, Carman CV (2012). Mesenchymal stem cells transmigrate between and directly through tumor necrosis factor-α-activated endothelial cells via both leukocyte-like and novel mechanisms. Stem Cells.

[134] Senst C, Nazari-Shafti T, Kruger S, Zu Bentrup KH, Dupin CL, Chaffin AE, Srivastav SK, Wörner PM, Abdel-Mageed AB, Alt EU (2013). Prospective dual role of mesenchymal stem cells in breast tumor microenvironment. Breast Cancer Res Treat.

[135] Lin G, Yang R, Banie L, Wang G, Ning H, Li LC, Lue TF, Lin CS (2010). Effects of transplantation of adipose tissue-derived stem cells on prostate tumor. Prostate.

[136] Gao H, Priebe W, Glod J, Banerjee D (2009). Activation of signal transducers and activators of transcription 3 and focal adhesion kinase by stromal cell-derived factor 1 is required for migration of human mesenchymal stem cells in response to tumor cell-conditioned medium. Stem Cells.

[137] Dwyer R, Potter-Beirne S, Harrington K, Lowery A, Hennessy E, Murphy J, Barry F, O'Brien T, Kerin M (2007). Monocyte chemotactic protein-1 secreted by primary breast tumors stimulates migration of mesenchymal stem cells. Clin Cancer Res.

[138] Goldstein RH, Reagan MR, Anderson K, Kaplan DL, Rosenblatt M (2010). Human bone marrow–derived MSCs can home to orthotopic breast cancer tumors and promote bone metastasis. Can Res.

[139] Chaturvedi P, Gilkes DM, Wong CCL, Luo W, Zhang H, Wei H, Takano N, Schito L, Levchenko A, Semenza GL (2012). Hypoxia-inducible factor–dependent breast cancer–mesenchymal stem cell bidirectional signaling promotes metastasis. J Clin Invest.

[140] Coffelt SB, Marini FC, Watson K, Zwezdaryk KJ, Dembinski JL, LaMarca HL, Tomchuck SL, Zu Bentrup KH, Danka ES, Henkle SL (2009). The pro-inflammatory peptide LL-37 promotes ovarian tumor progression through recruitment of multipotent mesenchymal stromal cells. Proc Natl Acad Sci.

[141] Luo J, Lee SO, Liang L, Huang C, Li L, Wen S, Chang C (2014). Infiltrating bone marrow mesenchymal stem cells increase prostate cancer stem cell population and metastatic ability via secreting cytokines to suppress androgen receptor signaling. Oncogene.

[142] Escobar P, Bouclier C, Serret J, Bièche I, Brigitte M, Caicedo A, Sanchez E, Vacher S, Vignais M-L, Bourin P (2015). IL-1β produced by aggressive breast cancer cells is one of the factors that dictate their interactions with mesenchymal stem cells through chemokine production. Oncotarget.

[143] Li W, Zhou Y, Yang J, Zhang X, Zhang H, Zhang T, Zhao S, Zheng P, Huo J, Wu H (2015). Gastric cancer-derived mesenchymal stem cells prompt gastric cancer progression through secretion of interleukin-8. J Exp Clin Cancer Res.

[144] Makinoshima H, Dezawa M (2009). Pancreatic cancer cells activate CCL5 expression in mesenchymal stromal cells through the insulin-like growth factor-I pathway. FEBS Lett.

[145] Barcellos-de-Souza P, Comito G, Pons-Segura C, Taddei ML, Gori V, Becherucci V, Bambi F, Margheri F, Laurenzana A, Del Rosso M (2016). Mesenchymal stem cells are recruited and activated into carcinoma-associated fibroblasts by prostate cancer microenvironment-derived TGF-β1. Stem Cells.

[146] Berger L, Shamai Y, Skorecki KL, Tzukerman M (2016). Tumor specific recruitment and reprogramming of mesenchymal stem cells in tumorigenesis. Stem Cells.

[147] Li H-J, Reinhardt F, Herschman HR, Weinberg RA (2012). Cancer-stimulated mesenchymal stem cells create a carcinoma stem cell niche via prostaglandin E2 signaling. Cancer Discov.

[148] Wu X-B, Liu Y, Wang G-H, Xu X, Cai Y, Wang H-Y, Li Y-Q, Meng H-F, Dai F, Jin J-D (2016). Mesenchymal stem cells promote colorectal cancer progression through AMPK/mTOR-mediated NF-κB activation. Sci Rep.

